# Pre-existing Symptoms and Healthcare Utilization Prior to Diagnosis of Neuroendocrine Tumors: A SEER-Medicare Database Study

**DOI:** 10.1038/s41598-018-35340-4

**Published:** 2018-11-15

**Authors:** C. Shen, A. Dasari, Y. Xu, S. Zhou, D. Gu, Y. Chu, D. M. Halperin, Y. T. Shih, J. C. Yao

**Affiliations:** 1 Department of Health Services Research, The University of Texas MD Anderson Cancer Center, Houston, USA; 2 Department of Biostatistics, The University of Texas MD Anderson Cancer Center, Houston, USA; 3 Department of Gastrointestinal Medical Oncology, The University of Texas MD Anderson Cancer Center, Houston, USA

## Abstract

The incidence and prevalence of neuroendocrine tumors (NETs) are continually increasing. While it is known that NET symptoms often predate diagnosis, their prevalence and impact on resource utilization and costs are largely unknown. We identified 9,319 elderly patients diagnosed with NETs between 1/2003 and 12/2011 from the Surveillance, Epidemiology and End Results (SEER)-Medicare. We examined the patients’ conditions potentially associated with NET, resource utilization and costs during the year before diagnosis. We found that NET patients were more likely to have diagnoses of hypertension (63.8% vs. 53.3%), abdominal pain (22.2% vs. 7.6%), heart failure (11.7% vs. 8.0%), diarrhea (5.8% vs. 1.8%), peripheral edema (5.4% vs. 3.8%) and irritable bowel syndrome (1.2% vs. 0.5%) compared to the non-cancer control group. They also had much higher resource utilization including number of outpatient visits (mean: 22.1 vs. 17.2), percentage with ER visits (20.9% vs. 11.6%), and hospitalizations (28.4% vs. 17.0%). Similarly, NET patients incurred significantly higher total (mean: $14602 vs. $9464), outpatient (mean: $5987 vs. $4253), and inpatient costs (mean: $8615 vs. $5211). This first population-based study on the pre-diagnosis symptoms and healthcare utilization found that NET patients were more likely to have certain conditions and incur higher resource utilizations and costs.

## Introduction

Neuroendocrine tumors (NETs), although traditionally thought to be rare, have increased in incidence by nearly 7-fold since 1973 and are now the second most commonly prevalent gastrointestinal malignancy after colorectal cancer, with an estimated 20-year limited duration prevalence of over 170,000 based on a recent study using Surveillance, Epidemiology, and End Results (SEER) registry data^1^. Neuroendocrine tumors are often diagnosed incidentally or when patients present with symptoms related to hormone production and/or tumor burden. However, the diagnosis even in those with symptoms is often delayed^2–4^. This could be related to potentially vague or non-specific symptoms leading to misdiagnoses, controversies in the diagnostic criteria and classification of NETs hindering accurate diagnosis, lack of experience with NETs amongst physicians, and inadequate access to sensitive tests such as somatostatin scintigraphy or NET specialty centers^2–4^. All these factors likely play a role in the delay of diagnosis of NETs. In a global online survey of over 1900 patients with NETs, the mean reported time from first symptom onset to diagnosis was 52 months with nearly 30% of responders reporting waiting 5 years or more for a formal NET diagnosis^5^. In this survey, patients reported seeing a mean of 6.2 health care providers across a mean of 11.8 visits before receiving their NET diagnosis^5^. While informative, these studies are hindered by important drawbacks such as selection and recall biases due to their study designs, and a population-based study can potentially overcome these limitations. Furthermore, prior studies have not analyzed the specialties of health care providers that NET patients see prior to diagnosis – this would be important to define the “pathway” to diagnosis for NET patients and identify areas where interventions may lead to diagnostic improvement. Finally, the implications of delayed diagnosis on health care resource utilization and related costs have not been explored yet.

The objectives of our U.S. population-based study were to examine the presence of common pre-existing symptoms, define the physician specialties involved, and estimate health care costs during the one year prior to diagnosis of NET among elderly patients.

## Materials and Methods

### Data Source

The data sources we used in the study included the Surveillance, Epidemiology, and End Results (SEER) registry data from the National Cancer Institute (NCI) linked with Medicare claims data and the American Medical Association (AMA) Physician Masterfile data^6^. The SEER cancer registry data include both clinical information (e.g., tumor characteristics) and patient demographics on cancer patients. The SEER registries cover approximately 28% of the U.S. population^7^. The linkage to Medicare claims data and the AMA Masterfile further enriches the data. The linkage to Medicare data adds information on the health care encounters that patients had both before and after cancer diagnosis and therefore allows us to identify patients’ medical conditions through International Classification of Diseases 9th Revision (ICD-9), Current Procedural Terminology (CPT), and Healthcare Common Procedure Coding System (HCPCS) codes. Further linkage with the AMA Masterfile allows us to capture physician characteristics such as specialty for each patient visit.

### Study Cohort

We included 9319 NET patients aged over 65 diagnosed between January 1, 2003 and December 31, 2011 from the SEER-Medicare database. The Medicare insurance program covers mainly people above 65 years old in the U.S., therefore we focused on this age group in this study. We identified NET patients of bronchopulomary or gastroenteropancreatic origin via International Classification of Diseases for Oncology, 3rd Edition (ICD-O-3) codes including: 8150, 8151, 8152, 8153, 8154, 8155, 8156, 8157, 8240, 8241, 8242, 8243, 8244, 8245, 8246, and 8249. Small cell and large cell neuroendocrine carcinoma of the lung, pheochromocytoma, paraganglioma and medullary carcinoma of the thyroid were not included. We required the patients in our study to have continuous enrollment in Medicare Parts A and B and no health maintenance organization (HMO) coverage during the 12 months before the NET diagnosis month so as to ensure complete claims information to identify health care encounters during this time frame.

### Identification of Potentially Relevant Conditions

We used literature review to identify symptoms commonly associated with NETs, and we considered patients to have had the relevant conditions if they had at least two indicative claims based on the ICD-9 codes during the one year before NET diagnosis^5^. The detailed list of codes used to identify conditions is provided in Supplementary Table 1.

### Physician Specialties

We captured the specialties of the physicians that the patients visited using the primary specialty information on the physicians in the AMA Masterfile. The specialties we considered included: primary, radiology, cardiovascular, emergency, gastroenterology, surgery, oncology, endocrinology, rheumatology and psychiatry. The detailed list of AMA primary specialty codes used to identify physician specialties is also provided in Supplementary Table 1.

### Resource Utilization and Costs of Care

We adopted a payer’s perspective and examined costs of care based on Medicare payment amount. We examined three types of costs: total costs, inpatients costs and outpatient costs. We studied the average monthly Medicare payment amount normalized to 2016 dollars based on the medical care services consumer price index^8^.

### Patient Characteristics

We included demographic and tumor characteristics in this study. The demographic characteristics that we used to identify a non-cancer control group were birth year, gender [male vs. female], race/ethnicity [non-Hispanic white, non-Hispanic black, Hispanics or all others], and region [Northeast, West, Midwest, South]. We provide descriptive tumor characteristics including tumor stage [localized, regional, distant, unstaged or unknown], primary cancer site [colon or rectum; small intestine, appendix or cecum; pancreas; lung, bronchus, larynx, trachea and other respiratory organ, and all others], and histology grade [grade I, grade II, grade III–IV, mixed histology grade, and unknown]. We would like to note that the histology grading system used in this paper follows SEER registry classification of carcinomas based on their morphology rather than the WHO classification based on proliferative indices such as Ki-67. Grade I would be analogous to well-differentiated, low grade; grade II to well-differentiated, intermediate grade; and grades III, IV to poorly differentiated or high grade in the SEER and WHO classifications respectively^9^. The mixed histology refers to mixed neuroendocrine and non-neuroendocrine neoplasms.

### Statistical Analyses

We used propensity score matching to identify 9319 comparable elderly patients from a non-cancer Medicare cohort. The propensity score was estimated using a logistic regression considering birth year, gender, race/ethnicity, and region. We compared the percentage of patients with symptoms that are common to NET disease between NET patients and the matched non-cancer control group. Percentages, chi-square tests and odds ratios (ORs) are provided. We also conducted subgroup analyses for the five most common conditions by stage, grade, and site. We evaluated the percentage of NET patients who visited doctors of specific specialties, and calculated the average number of visits for each specialty type among NET patients who had visits.

We compared health care costs between NET patients and the non-cancer control group including inpatient, outpatient and total costs using the Wilcoxon-Mann-Whitney test. We compared the number of outpatient visits using Wilcoxon-Mann-Whitney test, and compared the occurrence of emergency room (ER) admissions and hospitalizations using chi-square test. We focused on the 12 months prior to diagnosis; the month when the patient received the NET diagnosis was excluded from the analyses. We also conducted subgroup analyses for costs and healthcare utilizations by stage, grade, and site.

All statistical analyses were conducted in SAS Enterprise Guide 6.1 (SAS Institute, Cary NC). The Institutional Review Board at The University of Texas MD Anderson Cancer Center exempted this study for approval because all patients in the database had been de-identified.

## Results

Table 1 provides the comparison of NET patients with the non-cancer control group by age, gender, race and region. The two groups were very similar in terms of this demographic information. The p-values for the chi-square tests were above 0.77 for all four characteristics. The table also shows the tumor characteristics of the NET patients in this study. A large proportion (35%) of the patients had localized disease; more than half (53%) had grade I disease; 31% of the patients had lung, bronchus, larynx, trachea or other respiratory organs as their primary cancer site.

**Table 1:** Descriptive characteristics of NET patients and matched non-cancer controls.

	NET Patients	Non-cancer Controls	p-value
**Matched Variables**			
**Age**			0.9794
<70	2113 (22.67%)	2125 (22.80%)	
70–74	2470 (26.50%)	2447 (26.26%)	
75–79	2156 (23.14%)	2170 (23.29%)	
>=80	2580 (27.69%)	2577 (27.65%)	
**Gender**			0.7792
male	4119 (44.20%)	4100 (44.00%)	
female	5200 (55.80%)	5219 (56.00%)	
**Race**			0.8865
Non-Hispanic White	7407 (79.48%)	7411 (79.53%)	
Non-Hispanic Black	950 (10.19%)	963 (10.33%)	
Hispanic or Others	962 (10.32%)	945 (10.14%)	
**Region**			0.9855
Midwest	1112 (11.93%)	1098 (11.78%)	
Northeast	1897 (20.36%)	1912 (20.52%)	
South	2536 (27.21%)	2536 (27.21%)	
West	3774 (40.50%)	3773 (40.49%)	
**Tumor Characteristics**			
Stage			
Localized	3295 (35.36%)		
Regional	1619 (17.37%)		
Distant	2690 (28.87%)		
Unstaged or Unknown	1715 (18.40%)		
**Grade**			
Grade I	4911 (52.70%)		
Grade II	620 (6.65%)		
Grade III/IV	1431 (15.35%)		
Unknown	2107 (22.61%)		
Mixed Histology	250 (2.68%)		
**Site**			
Colon or rectum	1158 (12.43%)		
Lung, bronchus, larynx, trachea, or other respiratory organ	2867 (30.77%)		
Pancreas	692 (7.43%)		
Small intestine, appendix or cecum	2285 (24.52%)		
Other		2317 (24.86%)	

Note: For histology grades, grade I would be analogous to well-differentiated, low grade; grade II to well-differentiated, intermediate grade; and grades III, IV to poorly differentiated or high grade in the SEER and WHO classifications respectively. The mixed histology refers to mixed neuroendocrine and non-neuroendocrine neoplasms.

We found significant differences (p-value < 0.0001) between NET patients and non-cancer controls in hypertension (63.82% vs. 53.29%, OR = 1.55), abdominal pain (22.19% vs. 7.62%, OR = 3.46), heart failure (11.73% vs. 8.01%, OR = 1.53), diarrhea (5.81% vs. 1.78%, OR = 3.40) and peripheral edema (5.37% vs. 3.8%, OR = 1.44). We also found a significant difference in the frequency of irritable bowel syndrome (1.24% vs. 0.49%, OR = 2.54); we did not find significant differences in depression and anxiety between the two groups. In the subgroup analyses, we found overall higher odds of having these potential relevant symptoms across different stages, grades and sites. One exception is heart failure in patients with primary site at colon, rectum, or pancreas. This group of patients did not show significant difference in heart failure frequency from non-cancer controls. A few subgroups differences did not reach statistical difference for peripheral edema possibly due to the lower frequencies. Within the NET cohort, we did observe variation in the presence of symptoms and costs. However, such comparisons within the NET cohort need to be interpreted with substantial caution because of the observational nature of the current study. Due to the fact that the symptoms and costs were captured by claims in this observational study, patients who have higher number of encounters with the healthcare system (e.g. visit their doctors more often due to other chronic conditions or personal preference) are more likely to have their potentially relevant conditions recorded in their medical claims, incur higher costs; and they are also more likely to have their cancer detected earlier as they visit doctors more frequently. For example, we could observe patients with localized NET having more symptoms and incurring higher costs compared to patients with distant stage disease because of the above selection bias. Comparison of symptom presence by primary site is probably less prone to this issue, and we found significant differences by cancer site for all five symptoms. The results were overall as expected. Patients with small intestine, appendix or cecum as primary site were more likely to report abdominal pain and diarrhea; patients with colon, rectum or pancreas as primary site were less likely to report heart failure. The detailed results are presented in Table 2.

**Table 2:** Presence of symptoms in NET patients and non-cancer controls.

Symptoms	NET	Control	p-value	OR	95% CI
Hypertension	5947 (63.82%)	4966 (53.29%)	<0.0001	1.55	[1.46,1.64]
Abdominal Pain	2068 (22.19%)	710 (7.62%)	<0.0001	3.46	[3.16,3.79]
Heart Failure	1093 (11.73%)	746 (8.01%)	<0.0001	1.53	[1.38,1.68]
Diarrhea	541 (5.81%)	166 (1.78%)	<0.0001	3.4	[2.85,4.5]
Peripheral Edema	500 (5.37%)	354 (3.80%)	<0.0001	1.44	[1.25,1.65]
Depression	498 (5.34%)	460 (4.94%)	0.2075	1.09	[0.96,1.24]
Anxiety	279 (2.99%)	247 (2.65%)	0.157	1.13	[0.95,1.34]
Irritable Bowel Syndrome	116 (1.24%)	46 (0.49%)	<0.0001	2.54	[1.8,3.58]
Flushing	Masked*	Masked*	0.2748	1.67	[0.4,6.98]
**Subgroup Analyses by Stage**					
Hypertension					
Localized	2206 (66.95%)	1787 (54.23%)	<0.0001	1.71	[1.55,1.89]
Regional	1023 (63.19%)	850 (52.50%)	<0.0001	1.55	[1.35,1.79]
Distant	1586 (58.96%)	1373 (51.04%)	<0.0001	1.38	[1.24,1.54]
Abdominal Pain					
Localized	654 (19.85%)	256 (7.77%)	<0.0001	2.94	[2.52,3.43]
Regional	459 (28.35%)	113 (6.98%)	<0.0001	5.27	[4.23,6.57]
Distant	560 (20.82%)	204 (7.58%)	<0.0001	3.2	[2.7,3.8]
Heart Failure					
Localized	378 (11.47%)	249 (7.56%)	<0.0001	1.59	[1.34,1.88]
Regional	165 (10.19%)	130 (8.03%)	0.0326	1.3	[1.02,1.65]
Distant	299 (11.12%)	223 (8.29%)	0.0005	1.38	[1.15,1.66]
Diarrhea					
Localized	145 (4.40%)	59 (1.79%)	<0.0001	2.52	[1.86,3.43]
Regional	103 (6.36%)	23 (1.42%)	<0.0001	4.71	[2.98,7.45]
Distant	165 (6.13%)	50 (1.86%)	<0.0001	3.45	[2.5,4.76]
Peripheral Edema					
Localized	180 (5.46%)	143 (4.34%)	0.0348	1.27	[1.01,1.6]
Regional	64 (3.95%)	48 (2.96%)	0.1239	1.35	[0.92,1.97]
Distant	135 (5.02%)	91 (3.38%)	0.0028	1.51	[1.15,1.98]
**Subgroup Analyses by Grade**					
Hypertension					
Grade I	3274 (66.67%)	2658 (54.12%)	<0.0001	1.7	[1.56,1.84]
Grade II	392 (63.23%)	342 (55.16%)	0.0039	1.4	[1.11,1.75]
Grade III/IV	854 (59.68%)	712 (49.76%)	<0.0001	1.49	[1.29,1.73]
Mixed Histology	155 (62.00%)	129 (51.60%)	0.001	1.53	[1.07,2.19]
Abdominal Pain					
Grade I	1262 (25.70%)	381 (7.76%)	<0.0001	4.11	[3.64,4.65]
Grade II	134 (21.61%)	42 (6.77%)	<0.0001	3.79	[2.63,5.48]
Grade III/IV	215 (15.02%)	114 (7.97%)	<0.0001	2.04	[1.61,2.6]
Mixed Histology	73 (29.20%)	17 (6.80%)	0.0001	5.65	[3.22,9.92]
Heart Failure					
Grade I	572 (11.65%)	381 (7.76%)	<0.0001	1.57	[1.37,1.8]
Grade II	60 (9.68%)	37 (5.97%)	0.015	1.69	[1.1,2.58]
Grade III/IV	175 (12.23%)	126 (8.81%)	0.0028	1.44	[1.13,1.84]
Mixed Histology	23 (9.20%)	21 (8.40%)	0.0002	1.1	[0.6,2.05]
Diarrhea					
Grade I	334 (6.80%)	85 (1.73%)	<0.0001	4.14	[3.25,5.28]
Grade II	38 (6.13%)	Masked*	<0.0001	3.98	[1.97,8.07]
Grade III/IV	43 (3.00%)	19 (1.33%)	0.0021	2.3	[1.34,3.97]
Mixed Histology	20 (8.00%)	Masked*	0.2443	3.54	[1.4,8.96]
Peripheral Edema					
Grade I	278 (5.66%)	192 (3.91%)	<0.0001	1.47	[1.22,1.78]
Grade II	29 (4.68%)	22 (3.55%)	0.3168	1.33	[0.76,2.35]
Grade III/IV	54 (3.77%)	55 (3.84%)	0.9222	0.98	[0.67,1.44]
Mixed Histology	17 (6.80%)	Masked*	0.678	2.97	[1.15,7.66]
**Subgroup Analyses By Site**					
Hypertension					
Colon, rectum	697 (60.19%)	605 (52.25%)	0.0001	1.38	[1.17,1.63]
Lung, bronchus, larynx, trachea, other respiratory organ	1761 (61.42%)	1504 (52.46%)	<0.0001	1.44	[1.3,1.6]
Pancreas	444 (64.16%)	358 (51.73%)	<0.0001	1.67	[1.35,2.07]
Small intestine, appendix, cecum	1508 (66.00%)	1238 (54.18%)	<0.0001	1.64	[1.46,1.85]
Other	1537 (66.34%)	1261 (54.42%)	<0.0001	1.65	[1.47,1.86]
Abdominal Pain					
Colon, rectum	213 (18.39%)	103 (8.89%)	<0.0001	2.31	[1.8,2.97]
Lung, bronchus, larynx, trachea, other respiratory organ	347 (12.10%)	211 (7.36%)	<0.0001	1.73	[1.45,2.07]
Pancreas	219 (31.65%)	48 (6.94%)	<0.0001	6.21	[4.45,8.68]
Small intestine, appendix, cecum	768 (33.61%)	170 (7.44%)	<0.0001	6.3	[5.27,7.53]
Other	521 (22.49%)	178 (7.68%)	<0.0001	3.49	[2.91,4.18]
Heart Failure					
Colon, rectum	100 (8.64%)	98 (8.46%)	0.2765	1.02	[0.76,1.37]
Lung, bronchus, larynx, trachea, other respiratory organ	338 (11.79%)	210 (7.32%)	<0.0001	1.69	[1.41,2.03]
Pancreas	63 (9.10%)	63 (9.10%)	1	1	[0.69,1.44]
Small intestine, appendix, cecum	273 (11.95%)	174 (7.61%)	<0.0001	1.65	[1.35,2.01]
Other	319 (13.77%)	201 (8.68%)	<0.0001	1.68	[1.39,2.03]
Diarrhea					
Colon, rectum	43 (3.71%)	25 (2.16%)	0.0002	1.75	[1.06,2.88]
Lung, bronchus, larynx, trachea, other respiratory organ	75 (2.62%)	54 (1.88%)	0.0615	1.4	[0.98,1.99]
Pancreas	44 (6.36%)	13 (1.88%)	<0.0001	3.55	[1.89,6.64]
Small intestine, appendix, cecum	193 (8.45%)	30 (1.31%)	<0.0001	6.93	[4.7,10.23]
Other	186 (8.03%)	44 (1.90%)	<0.0001	4.51	[3.23,6.3]
Peripheral Edema					
Colon, rectum	42 (3.63%)	39 (3.37%)	0.186	1.08	[0.69,1.68]
Lung, bronchus, larynx, trachea, other respiratory organ	147 (5.13%)	98 (3.42%)	0.0014	1.53	[1.18,1.98]
Pancreas	42 (6.07%)	28 (4.05%)	0.0859	1.53	[0.94,2.5]
Small intestine, appendix, cecum	123 (5.38%)	85 (3.72%)	0.007	1.47	[1.11,1.95]
Other	146 (6.30%)	104 (4.49%)	0.0063	1.43	[1.11,1.85]

*Masked per SEER-Medicare user agreement for confidentiality.

OR: Odds ratio.

Note: For histology grades, grade I would be analogous to well-differentiated, low grade; grade II to well-differentiated, intermediate grade; and grades III, IV to poorly differentiated or high grade in the SEER and WHO classifications respectively. The mixed histology refers to mixed neuroendocrine and non-neuroendocrine neoplasms.

**Figure 1:**
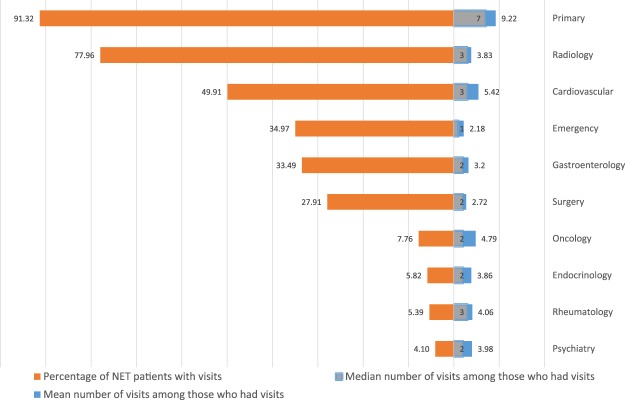
Patterns of physician visits during the 12 months before diagnosis among NET patients (10 most common specialties).

In Fig. 1, we show the patterns of visits to the 10 most common physician specialties to which NET patients sought treatment during the 12 months before diagnosis among NET patients. We found that the three most frequently visited specialties were primary care, radiology and cardiovascular physicians, with 91.32%, 77.96% and 49.91% of NET patients visiting, respectively. The next three most visited specialties were emergency (34.97%), gastroenterology (33.49%), and surgery (27.91%). The last four of the ten most visited specialties had a much lower percentage of patients with visits; the percentages ranged from 4.1% for psychiatry to 7.76% for oncology. Figure 1 also demonstrates the mean and median number of visits among the NET patients who had visited the corresponding specialties. As expected, the mean and median number of visits for primary care was the highest at 9.22 and 7, respectively, followed by cardiovascular specialty to which patients paid 5.42 visits on average with a median of 3. Although the percentage of patients visiting an oncologist was low at 7.76%, the number of visits was high (mean of 4.79 and median of 2).

Table 3 provides resource utilization during the 12 months before diagnosis comparing NET patients and non-cancer controls. We found significant differences (p-value < 0.0001) between NET patients and non-cancer controls in all three types of costs: total (mean: $14602.18 vs. $9463.73), outpatient (mean: $5987.17 vs. $4252.91) and inpatient (mean: $8615.01 vs. $5210.82). Further, we observed significant differences (p-value < 0.0001) in the number of outpatients visits (mean: 22.13 vs. 17.22), the occurrence of ER admissions (20.87% vs. 11.57%) and hospitalizations (28.40% vs. 17.01%) between NET and control individuals. We found similar results in the subgroup analyses showing significant differences in costs and resource utilizations across stages, grades, and sites.

**Table 3:** Resource utilization and costs during 12 months before diagnosis comparing NET patients and non-cancer controls.

	NET Patients			Non-cancer Controls			
	Mean	SD	Median	Mean	SD	Median	p-value
**Total costs**	14602.18	29165	5063.42	9463.73	22264.8	2316.17	<0.0001
**Outpatient costs**	5987.17	8269.78	3732.39	4252.91	7643.91	2047.11	<0.0001
**Inpatient costs**	8615.01	25874.8	0	5210.82	18639.9	0	<0.0001
**Number of outpatient visits**	22.13	16.72	18	17.22	15.58	13	<0.0001
	**Frequency**	**Percentage**		**Frequency**	**Percentage**		**p-value**
**Having any ER Admissions**	1945	20.87		1078	11.57		<0.0001
**Having any Hospitalizations**	2647	28.4		1585	17.01		<0.0001
***Subgroup Analyses by Stage***							
**Total costs**	**Mean**	**SD**	**Median**	**Mean**	**SD**	**Median**	**p-value**
Localized	15842.25	32437	5454.97	9704.82	22571.2	2206	<0.0001
Regional	14770.22	27633.5	5751.42	8881.38	20000.3	2163.84	<0.0001
Distant	12235.58	23846.7	4149.37	9546.25	24267.8	2390.89	<0.0001
**Outpatient costs**							
Localized	6574.61	8943.33	4215.15	4296.53	7396.88	2006.08	<0.0001
Regional	5774.95	6742.27	3910.87	4093.23	7340.44	1929.2	<0.0001
Distant	5248.64	7426.49	3197.03	4305.85	8245.13	2105.83	<0.0001
**Inpatient costs**							
Localized	9267.64	28799.5	0	5408.29	18622.8	0	<0.0001
Regional	8995.27	24989.8	0	4788.15	16647	0	<0.0001
Distant	6986.94	20990.7	0	5240.4	20698.9	0	<0.0001
**Number of outpatient visits**							
Localized	6574.61	8943.33	4215.15	4296.53	7396.88	2006.08	<0.0001
Regional	5774.95	6742.27	3910.87	4093.23	7340.44	1929.2	<0.0001
Distant	5248.64	7426.49	3197.03	4305.85	8245.13	2105.83	<0.0001
**Having any ER Admissions**	**Frequency**	**Percentage**		**Frequency**	**Percentage**		**p-value**
Localized	943	28.62%		556	16.87%		<0.0001
Regional	376	23.33%		1759	10.81%		<0.0001
Distant	495	18.40%		302	11.23%		<0.0001
**Having any Hospitalizations**							
Localized	679	20.61%		378	11.47%		<0.0001
Regional	493	30.45%		270	16.68%		<0.0001
Distant	679	25.24%		444	16.51%		<0.0001
***Subgroup Analyses by Grade***							
**Total costs**	**Mean**	**SD**	**Median**	**Mean**	**SD**	**Median**	**p-value**
Grade I	15534.43	31283.6	5463.53	9439.16	21488.4	2280.65	<0.0001
Grade II	11908.49	23901.5	4904.39	9217.39	21665.6	2167.1	<0.0001
Grade III/IV	13622.75	24333.5	4601.54	9709.22	20913.9	2585.37	<0.0001
Mixed Histology	14685.9	27043.8	4407.28	8961.7	23501.7	2612.84	<0.0001
**Outpatient costs**							
Grade I	6284.43	8456.3	4082.87	4311.32	7760.33	2031.5	<0.0001
Grade II	5692.42	6098.91	3922.06	4218.26	7028.03	2006.76	<0.0001
Grade III/IV	5328.57	7489.13	3309.24	4143.71	6414.61	2073.92	<0.0001
Mixed Histology	5839.8	7714.53	3470.46	3904.48	5311.84	2307.47	0.0007
**Inpatient costs**							
Grade I	9250	27919.2	0	5127.84	17325.2	0	<0.0001
Grade II	6216.07	22007.7	0	4999.12	18207.7	0	0.0001
Grade III/IV	8294.18	21551.9	0	5565.51	18291.3	0	<0.0001
Mixed Histology	8846.11	23244.5	0	5057.22	21805	0	<0.0001
**Number of outpatient visits**							
Grade I	22.83	16.65	19	17.33	15.94	13	<0.0001
Grade II	22.53	15.86	19	16.87	14.47	14	<0.0001
Grade III/IV	20.53	16.77	17	17.07	14.92	14	<0.0001
Mixed Histology	22.52	17.47	18	17.46	14.53	14	0.0009
**Having any ER Admissions**	**Frequency**	**Percentage**		**Frequency**	**Percentage**		**p-value**
Grade I	1050	21.38%		579	11.79%		<0.0001
Grade II	102	16.45%		65	10.48%		0.0021
Grade III/IV	292	20.41%		163	11.39%		<0.0001
Mixed Histology	61	24.40%		29	11.60%		0.0002
**Having any Hospitalizations**							
Grade I	1422	28.96%		853	17.37%		<0.0001
Grade II	152	24.52%		92	14.84%		<0.0001
Grade III/IV	400	27.95%		251	17.54%		<0.0001
Mixed Histology	73	29.20%		36	14.40%		<0.0001
***Subgroup Analyses by Site***							
**Total costs**	**Mean**	**SD**	**Median**	**Mean**	**SD**	**Median**	**p-value**
Colon, rectum	11656.11	25519.3	3175.43	9260.24	19918	2199.49	<0.0001
Lung, bronchus, larynx, trachea, other respiratory organ	13817.3	27719.6	5461.51	9030.99	22961	2202.63	<0.0001
Pancreas	14607.99	26877	5445.99	10850.47	28110.9	2619.58	<0.0001
Small intestine, appendix, cecum	16836.94	32798	5824.86	9537.66	21613.9	2214.08	<0.0001
Other	14840.14	29318.4	5014.15	9613.82	21130.8	2554.67	<0.0001
**Outpatient costs**							
Colon, rectum	4995.01	8383.72	2494.16	4254.25	6510.08	1951.85	<0.0001
Lung, bronchus, larynx, trachea, other respiratory organ	5970.99	7563.28	4140.5	4036.67	7894.94	1982.85	<0.0001
Pancreas	6483.69	8718.97	4160.01	4817.85	8276.41	2326.79	<0.0001
Small intestine, appendix, cecum	6158.14	8039.34	3874.47	4229.38	7380.68	1972.15	<0.0001
Other	6186.15	9066.24	3700.41	4374.28)	7902.39	2179.35	<0.0001
**Inpatient costs**							
Colon, rectum	6661.1	21156.9	0	5006	16885.4	0	0.0046
Lung, bronchus, larynx, trachea, other respiratory organ	7846.31	25075	0	4994.32	19142.8	0	<0.0001
Pancreas	8124.3	23190.4	0	6032.61	24288.6	0	<0.0001
Small intestine, appendix, cecum	10678.81	29735.7	0	5308.28	17890.6	0	<0.0001
Other	8653.99	25545.8	0	5239.54	17607.6	0	<0.0001
**Number of outpatient visits**							
Colon, rectum	18.64	15.27	15	17.11	16.83	13	0.0001
Lung, bronchus, larynx, trachea, other respiratory organ	22.27	16.69	19	16.82	15.42	13	<0.0001
Pancreas	23.73	17.34	20	18.15	15.78	14	<0.0001
Small intestine, appendix, cecum	22.91	16.88	19	17.19	15.17	13	<0.0001
Other	22.47	16.87	19	17.53	15.45	14	<0.0001
**Having any ER Admissions**	**Frequency**	**Percentage**		**Frequency**	**Percentage**		**p-value**
Colon, rectum	192	16.58%		125	10.79%		0.0036
Lung, bronchus, larynx, trachea, other respiratory organ	570	19.88%		320	11.16%		<0.0001
Pancreas	126	18.21%		82	11.85%		0.0009
Small intestine, appendix, cecum	577	25.25%		264	11.55%		<0.0001
Other	480	20.72%		2879	12.39%		<0.0001
**Having any Hospitalizations**							
Colon, rectum	259	22.37%		203	17.53%		0.0036
Lung, bronchus, larynx, trachea, other respiratory organ	790	27.55%		460	16.04%		<0.0001
Pancreas	194	28.03%		122	17.63%		<0.0001
Small intestine, appendix, cecum	740	32.39%		395	17.29%		<0.0001
Other	664	28.66%		405	17.48%		<0.0001

SD: standard deviation.

Note: For histology grades, grade I would be analogous to well-differentiated, low grade; grade II to well-differentiated, intermediate grade; and grades III, IV to poorly differentiated or high grade in the SEER and WHO classifications respectively. The mixed histology refers to mixed neuroendocrine and non-neuroendocrine neoplasms.

## Discussion

To the best of our knowledge, this is the first population-based study to examine potentially relevant pre-existing symptoms and resource utilization of patients, and associated health care costs, before NET diagnosis. Overall, we found that NET patients were more likely to have potentially relevant symptoms and increased health care encounters leading to much higher health care costs compared to the non-cancer controls.

We found that NET patients incurred much higher mean health care costs (approximately $5000) than the non-cancer control group during the 12 months before diagnosis, with around 70% of the cost difference coming from inpatient costs and 30% due to outpatient costs. Since surgeons were one of the common specialists seen by NET patients, we also examined whether surgeries contributed to increased inpatient costs from procedures and associated post-operative recovery. We calculated the costs related to surgery during the 12 months before diagnosis by adding up the costs from claims indicating surgery treatments. Indeed, we found that the magnitude of surgery cost difference between NET patients and controls was around $2400 and almost exclusively due to inpatient costs. For instance, it is likely that NET patients, due to unexplained symptoms such as chronic or recurrent abdominal pain, may undergo surgical exploration for workup and/or procedures such as cholecystectomy. In addition to the surgical costs, since NET patients have more ER visits, it is likely that these may have resulted in more inpatient admissions for workup and management of the causative symptoms. Higher outpatient costs for NET patients is also unsurprising given the higher number of outpatient and ER visits identified in our studies. Since our study was limited to 12 months prior to diagnosis and it has been established that NET patients have symptoms on an average for 4–5 years pre-diagnosis, it is very likely that the actual cost of delayed diagnosis of NETs is manifold higher. Therefore, it is imperative to identify potential strategies to facilitate earlier diagnosis of NETs.

We found the most common presenting symptoms of NET patients prior to diagnosis were hypertension and abdominal pain. Of note, symptoms of diarrhea and flushing, which are often associated with carcinoid syndrome, were not commonly reported. This is unsurprising since it is very likely that patients with these symptoms were misdiagnosed as having other conditions with similar symptoms such as irritable bowel syndrome, as demonstrated by prior studies^2–4^. When we examine the presence of the five most common potential symptoms in subgroups by stage, grade and site, we found overall higher odds of having these symptoms except heart failure in patients with primary site at colon, rectum or pancreas. This is probably because NET tumors from these locations typically do not produce hormones associated with carcinoid heart disease^10^. However it should be noted that ICD-9 coding in our data cannot reliably distinguish left from right heart failure. Also, left heart failure is a common health issue unrelated to NETs in older patients. The clinical manifestations of right heart failure specifically and costs involved over the entire clinical course of NETs need to be addressed in future studies.

The patterns of visits to physician specialties prior to diagnosis correlate with the symptoms, i.e., a very likely pathway to diagnosis of NETs is that patients present to their primary care physicians (the most common specialty visit in our study) and may go undiagnosed in spite of preliminary workup. These individuals are then likely to be referred to other specialties for further management of persistent symptoms including abdominal pain (gastroenterology & surgery), hypertension, peripheral edema (cardiology) and diarrhea (gastroenterology). A minority are likely referred to oncologists based on suspicion of cancer (e.g., abnormal mass identified on scans) prior to the diagnosis of NET, who then obtain a formal diagnosis and utilize oncology services, as evidenced by the small proportion of patients with oncology claims but with higher number of visits. All these specialty physicians likely undertake extensive workup including scans as reflected in the high number of radiology claims. Other studies in the literature have also found the pathways to diagnosis of cancer to be very complex in other cancer types such as breast, lung, and colorectal cancer^11–14^. Our analysis did not uncover a specific constellation of symptoms that could predict a diagnosis of NET. However, it is important to educate physicians, especially those involved in primary care, cardiology, gastroenterology, radiology and surgery to consider NET as a differential diagnosis in patients with multiple visits for recurrent or persistent abdominal pain especially when associated with other symptoms such as hypertension, diarrhea and heart failure. To increase their awareness and recognition of NETs, physicians, especially in these specialties should be educated both during their training and beyond regarding the typical presenting symptoms of NETs and the various modalities of diagnostic techniques for NETs including serum/urine biomarkers, general radiological tests such as computerized tomography, magnetic resonance imaging and the typical appearance of NETs on these scans and more specific tests such as somatostatin receptor scintigraphy and the more recent gallium 68 PET/CT^4,15^.

This study is based on SEER-Medicare data and therefore inherits the common limitations of observational studies. We only included patients at least 65 years and older with Medicare insurance due to the data limitations. It is possible that higher prevalence of potentially relevant pre-existing symptoms might be even more prominent among younger patients as their non-cancer counterparts have less comorbidities. As mentioned above, some symptoms may be underdiagnosed or misdiagnosed by physicians and therefore are not captured by claims information. Thus, the actual prevalence of potentially relevant pre-existing symptoms and the corresponding costs might be even higher. Nevertheless, as the first population-based study in the literature, this current study showed the convoluted pathway to the diagnosis of NETs that results in great financial costs. Future studies should focus on identifying strategies towards early diagnosis of NET patients.

## Electronic supplementary material

Supplementary Tables

## Data Availability

The data that support the findings of this study are disclosed in the paper. The raw data should be requested from the NCI, CMS, IMS and SEER Program.
