# Supplementary material for: Pre-existing Symptoms and Healthcare Utilization Prior to Diagnosis of Neuroendocrine Tumors: A SEER-Medicare Database Study
Source: Sci Rep. 2018 Nov 15;8:16863. doi: 10.1038/s41598-018-35340-4 (PMC6238007; doi:10.1038/s41598-018-35340-4)
Supplement: Supplementary file 1 — Supplementary Tables [file 41598_2018_35340_MOESM1_ESM.docx]

**Pre-existing Symptoms and Healthcare Utilization Prior to Diagnosis of Neuroendocrine Tumors: A SEER-Medicare Database Study**

C. Shen^1,2^, A. Dasari^3^, Y. Xu^1^, S. Zhou^2^, D. Gu^1^, Y. Chu^1^, D. M. Halperin^3^, Y. T. Shih^1^, J. C. Yao^3^

^1^Department of Health Services Research, The University of Texas MD Anderson Cancer Center, Houston, USA; ^2^Department of Biostatistics, The University of Texas MD Anderson Cancer Center, Houston, USA; ^3^Department of Gastrointestinal Medical Oncology, The University of Texas MD Anderson Cancer Center, Houston, USA

Supplementary Table 1. Codes for potentially relevant conditions and physician specialties

| **Conditions** | **ICD-9 code** |
| --- | --- |
| Diarrhea | 787.91,564.5 |
| Flushing | 782.62 |
| Abdominal pain | 789.0 |
| Irritable bowel Syndrome | 564.1 |
| Hypertension | 401 |
| Heart failure | 428 |
| Peripheral edema | 782.3 |
| Depression | 293.83, 296.20, 296.21, 296.22 ,296.23, 296.24, 296.25, 296.26, 296.30, 296.31, 296.32, 296.33, 296.34, 296.35, 296.36, 300.4,311 |
| Anxiety | 293.84, 300.00, 300.01, 300.02, 300.09, 300.10, 300.20,300.21, 300.22, 300.23, 300.29, 300.3, 300.5, 300.89, 300.9 308.0, 308.1, 308.2,308.3, 308.4, 308.9, 309.81, 313.0,313.1, 313.21, 313.22, 313.3, 313.82, 313.83 |
| **Physician Specialties** | **AMA Primary Specialty Code** |
| Primary | "IFP" ,"Internal Medicine - Family Practice"  "IM", "Internal Medicine"  "IMA", "Internal Medicine/Anesthesiology"  "IMD", "Internal Medicine/Dermatology  "IMG", "Internal Medicine - Geriatrics"  "INM", "Internal Medicine / Nuclear Medicine"  "IPM", "Internal Medicine - Preventive Medicine"  "ISM", "Internal Medicine - Sports Med"  "MDG", "Internal Medicine / Medical Genetics"  "MEM", "Internal Medicine - Emergency Medicine"  "MN", "Internal Medicine - Neurology"  "MP", "Internal Medicine - Psychiatry"  "MPD", "Internal Medicine - Pediatrics"  "MPM", "Internal Medicine - Physical Medicine And Rehab"  "GP", "General Practice"  "FP", "Family Practice"  "FPG", "Family Practice/geriatric Med"  "FSM", "Family Prac/sports Medicine"  "SMI", "Sleep Medicine (Internal Medicine)" |
| Radiology | "AR", "Abdominal Radiology"  "CTR", "Cardiothoracic Radiology"  "DR", "Diagnostic Radiology"  "R", "Radiology" |
| Cardiovascular | "CD", "Cardiovascular Disease" |
| Emergency | "EFM", "Emergency Medicine/Family Medicine"  "EM", "Emergency Medicine"  "EMS", "Emergency Medical Services"  "ESM", "Emergency/sports Medicine"  "ETX", "Emergency Medical Toxicology"  "IEC", "IM/Emergency Medicine/Critical Care Medicine" |
| Gastroenterology | "GE", "Gastroenterology" |
| Surgery | "GS", "General Surgery"  "AS", "Abdominal Surgery"  "ASO", "Advanced Surgical Oncology"  "CRS", "Colon & Rectal Surgery"  "TRS", "Traumatic Surgery"  "TS", "Thoracic Surgery"  "TSI", "Thoracic Surgery - Integrated"  "SO", "Surgical Oncology" |
| Oncology | "ON", "Oncology"  "HO", "Hematology/oncology" |
| Endocrinology | "END", "Endocrinology" |
| Rheumatology | "RHU", "Neuroradiology" |
| Psychiatry | "P", "Psychiatry" |

| Supplementary Table 2. Healthcare costs related to surgery during 12 months before NET diagnosis | | | | |
| --- | --- | --- | --- | --- |
|  | NET patients | Noncancer Controls | P |  |
| **Whole Sample** | | | |  |
| **Total Costs - Surgery($)** | |  | <.0001 |  |
| Mean(SD) | 4374.67(17872.2) | 1963.67(10522.3) |  |  |
| **Inpatient Costs -Surgery($)** | |  | <.0001 |  |
| Mean(SD) | 4304.70(17840.8) | 1926.50(10490.0) |  |  |
| **Outpatient Costs - Surgery($)** | |  | <.0001 |  |
| Mean(SD) | 69.96( 751.88) | 37.16( 408.97) |  |  |
| **Percentage with positive surgery costs** | |  | <.0001 |  |
|  | 20.79% | 11.05% |  |  |
| **Subgroup with positive surgery costs** | | | |  |
| **Total Costs - Surgery($)** | |  | 0.0022 |  |
| Mean(SD) | 21046.74(34442.3) | 17766.43(26862.1) |  |  |
| Median | 10914.75 | 10085.47 |  |  |
| **Inpatient Costs -Surgery($)** | |  | 0.0037 |  |
| Mean(SD) | 20710.14(34525.6) | 17430.19(26943.8) |  |  |
| Median | 10750.88 | 9959.42 |  |  |
| **Outpatient Costs - Surgery($)** | |  | 0.0724 |  |
| Mean(SD) | 336.60(1622.08) | 336.23(1189.09) |  |  |
| Median | 0 | 0 |  |  |
